# Monoclonal Gammopathy of Neurological Significance: Toward an Integrated Hematologic–Neurologic Perspective—A Single-Center Retrospective Study

**DOI:** 10.3390/ijms27093847

**Published:** 2026-04-26

**Authors:** Sorina Badelita, Larisa Zidaru, Sinziana Barbu, Iulia Ursuleac, Mirela Draghici, Camelia Dobrea, Monica Popescu, Daniel Coriu

**Affiliations:** 1 Clinical Department I, Hematology, Fundeni Clinical Institute, 022328 Bucharest, Romania; sorinabadelita@gmail.com (S.B.); sinziana.baitan@yahoo.com (S.B.); iuliaursuleac@yahoo.com (I.U.); mirelaneuro@yahoo.com (M.D.); cameliadobrea@yahoo.com (C.D.); monica2982003@yahoo.com (M.P.); daniel_coriu@yahoo.com (D.C.); 2 Hematology Department, “Carol Davila” University of Medicine and Pharmacy, 050474 Bucharest, Romania

**Keywords:** MGCS, MGNS, MGUS, peripheral polyneuropathy, IgM, POEMS, cryoglobulinemia, real-world data

## Abstract

Monoclonal gammopathies of clinical significance (MGCSs) are entities in which a small hematological clone produces a monoclonal immunoglobulin capable of causing organ damage. Neurological involvement remains difficult to diagnose and treat, especially in the context of incidental monoclonal gammopathy of undetermined significance (MGUS)–peripheral neuropathy (PN) associations. We conducted a single-center retrospective study at Fundeni Clinical Institute, Bucharest, from January 2015 to December 2025. The reference population included 300 patients with MGUS. The diagnosis of MGNS was established clinically and/or electrophysiologically, with the exclusion of alternative causes of neuropathy. In total, 35 patients with MGNS were identified (prevalence 11.7%). Neuropathy was more common in IgM MGUS (36.7%) compared to IgG (15%), IgA (14.3%), or light chain MGUS (16.7%), with an increased risk for IgM (OR 3.27, *p* < 0.001). A total of 88.5% of patients received hematological treatment; neurological response was noted in the majority of treated patients. Mortality was 14.3%, and median OS was not reached. Our findings confirm the dissociation between low clonal load and the severity of organ involvement. IgM MGUS is associated with a significantly increased risk of neuropathy, supporting the need for systematic screening for MGUS in patients with PN and for a multidisciplinary approach.

## 1. Introduction

Monoclonal gammopathy of clinical significance (MGCS) is a recently introduced concept formalized in 2018, derived from monoclonal gammopathy of undetermined significance (MGUS) [1,2,3]. It implies the presence of a plasma cell or B-cell clone, generally small in size, capable of generating severe clinical manifestations through the biological properties of the secreted monoclonal immunoglobulin (Mo Ig) [1,2,3,4].

Unlike MGUS, MGCS is associated with organ damage attributable to Mo Ig, in the absence of standard diagnostic criteria for multiple myeloma (MM) or other lymphoproliferative disorders, thus representing a separate entity within the spectrum of monoclonal gammopathies [1,2,3].

The MGCS entity includes subtypes such as monoclonal gammopathy of renal significance (MGRS), neurological significance (MGNS), ocular significance (MGOS), cutaneous significance (MGSS) [1,2,3], and, more recently, thrombotic significance (MGTS) [5].

Each MGCS subtype can be found separately, representing a distinct type of disorder, or in association with other organ involvements [1].

As many subgroups within each MGCS category present as syndromes [1,2], recognizing them through clinical manifestations is a crucial step in the diagnosis and management of MGCS [1,2,3]. A defining feature of this group of disorders is the lack of correlation between the size of the hematologic clone and organ involvement, which is often severe and sometimes irreversible [1,3].

MGNS is part of a spectrum of disorders in which a monoclonal protein causes neurological manifestations, most commonly peripheral neuropathies, leading to a clinical picture characterized by muscle weakness, paresthesia, and pain [2,6,7,8]. This condition represents a real challenge, both in terms of diagnosis and treatment [6,7]. Diagnosis is difficult due to the heterogeneous clinical presentation and the frequent lack of a diagnostic biopsies, unlike other MGCS [7].

Because there is a relatively high prevalence of peripheral neuropathy (PN) in the MGUS subpopulation (4–10%), and because both neuropathy and MGUS increase in incidence with age, the possibility of a non-causal, coincidental relationship should always be considered [8,9,10]. This distinction is essential to avoid unnecessary and potentially toxic therapies [6,8,10].

There are several defined entities within the MGNS spectrum, differentiated by Mo Ig type, neuropathy pattern, disease progression, and laboratory tests, such as AL amyloidosis, POEMS syndrome, type I/II cryoglobulinemia, distal acquired polyneuropathy associated with monoclonal gammopathy (DADS-M), CANOMAD syndrome, or late-onset non-malignant myopathy (SLONM) [1,6,7,11].

POEMS syndrome is a multisystemic disorder in which monoclonal protein, most commonly lambda, causes cytokine dysregulation, particularly increased VEGF levels, resulting in neurological impairment and multiple systemic manifestations [1,11,12]. Its dominant feature is a sensory–motor demyelinating peripheral neuropathy [1,12].

A particular subset of MGCS is represented by MGCS associated with IgM MGUS [2], including cold agglutinin disease, type 1 and 2 cryoglobulinemia, IgM-associated neuropathies, IgM POEMS, acquired von Willebrand syndrome, etc. [13].

Cryoglobulinemia is divided into type I, which usually involves monoclonal IgG or IgM, often leading to vascular occlusion, and mixed cryoglobulinemia (type II/III), which is typically linked to autoimmune vasculitis [14,15].

Regarding treatment, the therapeutic strategy is guided by clinical symptoms rather than by the size of the hematologic clone [1,3,4,16] and varies according to the affected organ, the type of hematological clone, and the dominant pathophysiological mechanisms [1,2,3,4,16]. The main objective is to reduce monoclonal protein production while limiting and improving the lesions caused by it [1,3,4].

Treatment for those entities is far from standardized and usually requires a multidisciplinary approach [3,4,13,16,17]. Taking into account the isotype of the monoclonal protein involved, non-IgM entities are usually treated with specific multiple myeloma-directed therapies (immunomodulators, proteasome inhibitors, anti-CD38 antibodies), whereas IgM entities are mainly treated with an anti-CD20 approach [3,4,16]. In addition to clone-directed therapy, supportive therapy is essential in each category of MGCS. For example, in many categories of MGNS associated with demyelinating neuropathy, intravenous immunoglobulins (IVIG) and/or corticosteroid therapy [7] may be required to alleviate neurological symptoms [6,8,18].

In this article, we aim to analyze MGCS forms with neurological significance, focusing on the main aspects of diagnostic challenges and the principles of clone- and organ-focused treatment.

## 2. Results

### 2.1. Selection and Composition of the MGNS Cohort

**Figure 1:**
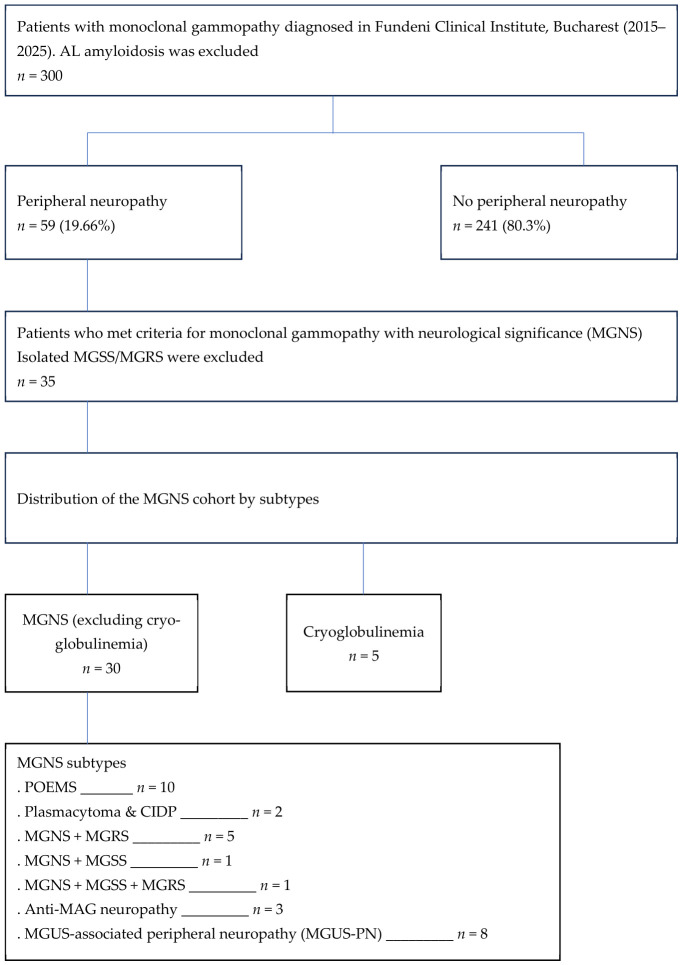
Patient selection and MGNS subtype distribution flow diagram at the Fundeni Clinical Institute cohort (2015–2025).

We conducted a retrospective study at the Fundeni Clinical Institute, Bucharest, between January 2015 and December 2025 and identified 35 patients who met the criteria for MGNS, corresponding to a prevalence of 11.7% within the overall MGUS population (Figure 1).

All patients were diagnosed and followed in the Hematology and Neurology Department of the Fundeni Clinical Institute in Bucharest. Our cohort included patients with MGNS, comprising specific subgroups such as POEMS syndrome, cryoglobulinemia, and cases with plasmacytoma associated with CIDP-like features, all of which belong to the broader spectrum entities of MGCS and are discussed separately (Figure 1).

Of the 35 patients, 30 were classified as having MGNS (excluding cryoglobulinemia), and five patients were classified as having cryoglobulinemia with neurological involvement (Figure 1).

### 2.2. Overall Prevalence of Neuropathy in MGUS

In addition to patients diagnosed with MGUS (*n* = 300), 24 patients were identified as having peripheral neuropathy considered incidental, corresponding to a prevalence of 8% in the MGUS cohort. The incidental association between MGUS and peripheral neuropathy was considered in the absence of sufficient evidence to support a direct causal relationship between monoclonal gammopathy and neurological impairment [8,9,19].

This consideration was applied when an alternative cause of neuropathy existed and/or when well-defined MGCS entities associated with neurological impairment, such as AL amyloidosis, cryoglobulinemia, and POEMS syndrome, were excluded, in the absence of additional clinical, biological, or histopathological evidence supporting a monoclonal protein-mediated nerve injury.

In this subgroup, alternative explanations for neuropathy included diabetes mellitus (*n* = 6), diabetes mellitus associated with Parkinson’s disease (*n* = 2), diabetes associated with significant vascular disease with a prior angioplasty and stenting (*n* = 1), disc herniation (*n* = 3), malignancies (*n* = 2), an autoimmune disease (*n* = 1), thyroid disease (*n* = 1), and spontaneous resolution without specific therapeutic intervention (*n* = 1).

An additional seven patients, almost all with IgG MGUS (6/7 patients), were classified as having a probable incidental association after exclusion of all the entities with a known or probable causal relationship between the monoclonal protein and neurologic involvement. Of these, three had negative nerve biopsies, and one had muscle biopsy findings suggestive of myositis, supporting an alternative autoimmune mechanism.

The Mo Ig subtype distribution among patients with incidental MGUS-associated neuropathy was as follows: 6 patients with IgM MGUS (25%), 17 with IgG MGUS (70%), and 1 with IgA MGUS (4.16%); the light chain subtype was not reported.

Overall, the total prevalence of PN in the MGUS cohort was 19.66% (59/300 patients) (Figure 1).

### 2.3. Association Between MGUS Subtype and Neuropathy

A statistically significant association between the presence of peripheral PN and MGUS subtype was demonstrated (χ^2^(3) = 14.347, *p* = 0.002). In patients with IgM MGUS, the prevalence of PN was significantly higher than in those with IgG, IgA, or light-chain MGUS (36.7% vs. 15%, 14.3%, and 16.7%), with Monte Carlo testing confirming statistical significance at *p* < 0.001.

### 2.4. Comparative Analysis of IgM vs. Non-IgM

Patients with IgM MGUS had 3.27-fold higher odds of neuropathy than those with non-IgM MGUS (OR = 3.265; 95% CI: 1.733–6.151; *p* < 0.001). This result was also confirmed by Fisher’s exact test (*p* < 0.001).

**Figure 2:**
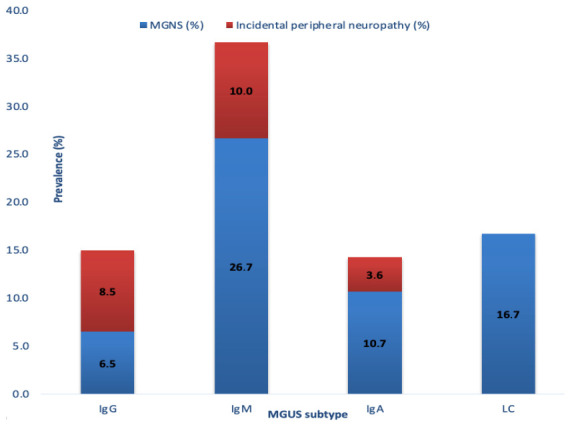
Stacked column chart illustrating the prevalence of MGNS (causal neuropathy) and incidental peripheral neuropathy within each MGUS subtype.

Further stratification according to causal neuropathy (MGNS) versus incidental neuropathy revealed distinct patterns between subtypes. In IgG MGUS, incidental neuropathy was slightly more frequent than MGNS (8.5% vs. 6.5%). In contrast, IgA MGNS appeared more frequent than incidental neuropathy (10.7% vs. 3.6%). In the IgM subgroup, MGNS clearly predominated over incidental neuropathy (26.7% vs. 10%) (Figure 2).

### 2.5. General Characteristics of the Cohort

The MGNS group included 10 patients with POEMS syndrome, 2 patients with plasmacytoma associated with chronic inflammatory demyelinating polyneuropathy (CIDP), 5 patients with MGNS + MGRS, 1 patient with MGNS + MGSS, 1 patient with MGNS + MGSS + MGRS, and 3 patients with anti-MAG neuropathy. The remaining eight patients were classified as MGNS based on the existence of MGUS and neurological involvement (MGUS-PN), Figure 1. The other five patients were part of the cryoglobulinemia group, all of whom had both neurological and renal involvement.

Only three patients (15%) were diagnosed with this anti-MAG neuropathy. However, anti-MAG antibody testing was not performed in more than half of the patients (11/20 patients, 55%). This proportion should be interpreted with caution because it underestimates the true frequency of anti-MAG neuropathy in our study.

Patients diagnosed with MGSS (*n* = 11, 31.42%) had a varied clinical presentation: 1 patient with clinical features of necrobiotic xanthogranulomatosis, 1 other patient with nonspecific erythema associated with livedo reticularis-like lesions. The remaining nine patients with skin involvement were included in the cryoglobulinemia subgroup (five patients, all of them with cryoglobulinemic vasculitis) or the POEMS syndrome subgroup (four patients), as detailed in the sections corresponding to each subgroup.

Renal impairment in patients diagnosed concomitantly or during the course of MGRS (11/35, 31.42%) consisted of: PGNMID (3/11 patients), immunotactoid glomerulonephritis (1/11 patients), light chain proximal crystalline tubulopathy (LCPT, 2/11 patients), and cryoglobulinemic glomerulonephritis (5/11 patients). All patients with cryoglobulinemia had renal involvement, with kidney biopsy confirming cryoglobulinemic glomerulonephritis.

Patient characteristics are presented in Table 1.

The median age for the entire cohort was 59 years (range 32–86 years), and 60% of patients were male.

In total, 31/35 patients (88.5%) received hematological treatment, which is discussed separately in each specific subchapter. Patients who did not receive treatment are either patients who refused or are undergoing investigations, as well as patients who were lost to follow-up. Eleven patients (31.42%) are still undergoing treatment at the time of analysis (January 2026).

From a hematological point of view, ORR (≥PR) was observed in 20 treated patients (4 CR, 4 PR, 12 VGPR). Seven patients recorded an SD response, and for four patients, this is currently being recorded or is unknown.

The hematological response was assessed according to the International Myeloma Working Group (IMWG) response criteria [20].

All patients with neuropathy received symptomatic/neurotrophic treatment, including alpha-lipoic acid and/or gabapentin. Furthermore, intravenous immunoglobulins were administered to 16 out of 35 patients, including 7 in the POEMS subgroup, 2 patients with CIDP-like neuropathy associated with plasmacytoma, and the remaining patients with MGNS and concurrent CIDP. However, the influence of these therapies on overall survival could not be definitively determined because of the study’s retrospective design, the cohorts’ heterogeneity, and the small number of patients.

Neurological response was present in 23/31 treated patients (19/23 patients with a favorable outcome and the remaining four patients with a stable outcome), consistent with hematological ORR.

**Figure 3:**
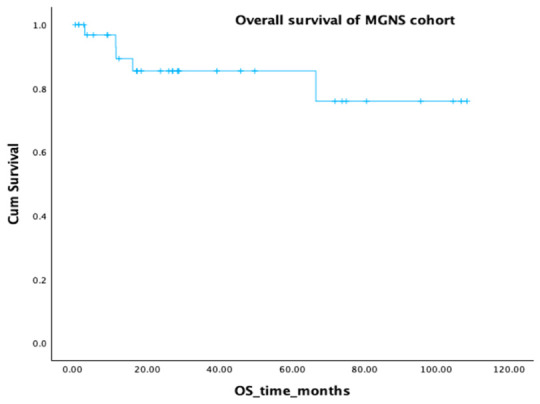
Kaplan–Meier overall survival in the entire group. Crosses indicate censored observations.

The median follow-up duration was 28.3 months (min–max: 0–108.5 months) and the median OS for the entire patient cohort was not reached in our retrospective analysis (Figure 3). The estimated mean OS was 90 months (95% CI, 75.7–104.5). The estimated OS at 24,48 and 60 months was approximately 85%, and at 72 months, approximately 76%. Given the median follow-up of 28.3 months, OS at this time point was also reported and was 85%.

**Figure 4:**
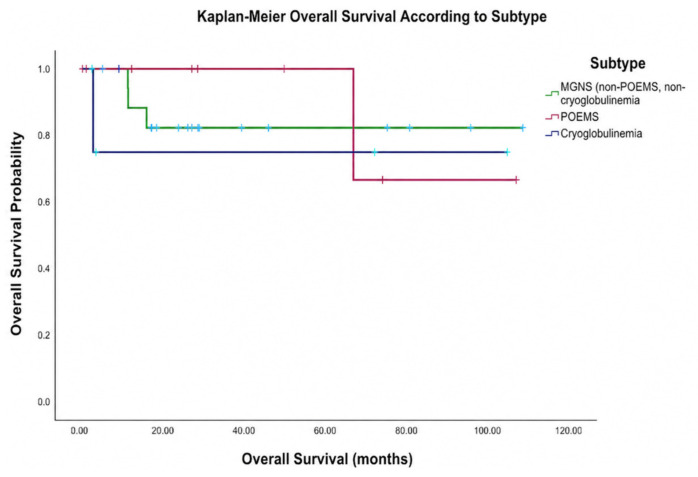
Kaplan–Meier analysis of overall survival according to subtype, including MGNS without POEMS or cryoglobulinemia, POEMS syndrome, and cryoglobulinemia. Cross symbols indicate censored observations.

No statistical difference in OS was observed among the MGNS (excluding POEMS and cryoglobulinemia), POEMS syndrome and cryoglobulinemia subgroups (Figure 4) with a log-rank *p* = 0.821.

Five deaths were reported during the study period, with a heterogeneous distribution of causes. One 77-year-old patient with multiple comorbidities and severe renal impairment due to MGRS died from a gastrointestinal infection during treatment (cycle 11 of treatment with lenalidomide and dexamethasone). Another 76-year-old patient, also with multiple comorbidities and severe renal impairment in the context of MGRS, died from infectious complications 5 months after the sixth cycle of rituximab, corticosteroid, and cyclophosphamide, in the setting of marked immunosuppression. A 57-year-old patient who had recently been exposed to rituximab-bendamustine developed severe neutropenia complicated by an infection and died of sepsis. Another death occurred in a 51-year-old patient with POEMS syndrome, without active hematologic treatment for approximately 3 years, due to pulmonary thromboembolism. An additional patient, aged 75, died of unknown cause.

### 2.6. Monoclonal Gammopathy with Neurological Significance (Excluding POEMS Syndrome and Cryoglobulinemia)

In the subgroup of patients with monoclonal gammopathy with neurological significance (*n* = 20 patients), excluding patients with POEMS syndrome or cryoglobulinemia, the median age at diagnosis was 68 years, with an equal distribution between sexes (50% M, 50% F). The median interval between the onset of symptoms and diagnosis was 7 months.

The plasma cell clone in bone marrow was predominant and found in 11 patients (55%), while the lymphoplasmacytic clone was present in 6 cases (30%), and the lymphocytic clone in 3 cases (15%), with a median hematological bone marrow infiltrate at 9%.

The Mo Ig type was IgG in 6 patients (30%), IgM in 12 patients (60%), and LC in 2 patients (10%), and the median value of the monoclonal component was 0.3 g/dl. The involved light chain type was kappa in 14 patients (70%) and lambda in only 6 patients (30%). The median dFLC was only 13 mg/L. Among MGNS IgM-positive patients (12/20 patients, 60%), the MYD88L265P mutation was negative in all patients tested (eight patients had a documented negative result, and in four cases, the analysis was not performed).

Electromyography tests were performed in 19/20 patients and showed that 9 patients (45%) had axonal neuropathy, 10 patients (50%) had features compatible with CIDP-like diagnosis, and only one patient (5%) with MGNS + MGRS has suggestive symptoms and will undergo this test soon. The sensory fiber involvement was present in five patients (25%).

Sensory fiber involvement alone was found in five patients (25%), and was mixed sensory–motor in 14 patients (70%). Half of the patients had clinical onsets in the lower limb, while the other half had concurrent upper and lower limb symptoms. Paresthesias were the most common symptoms and were present in all patients, whereas muscle weakness was observed in all 10 patients with a CIDP-like pattern.

Most patients (17/20 patients—85%) underwent treatment. Patients still undergoing investigation or those who refused treatment did not receive therapy at the time of analysis (January 2026). Five patients (25%) are currently undergoing treatment. A total of 9/10 patients diagnosed with CIDP also underwent intravenous immunoglobulin therapy.

Hematological treatment was chosen according to the Mo Ig isotype and the type of clone and is detailed in Table 2.

Hematological response was CR in 3 patients (15%), VGPR in 6 patients (30%), PR in 3 patients (15%), SD in 4 patients (20%) and 1 patient is awaiting response evaluation.

Hematological and clinical progression of the disease was recorded in two patients, both currently undergoing second-line treatment.

Neurological response was observed in 13 patients (65%) and consisted of improvement (12 patients) or stabilization (one patient) of clinical parameters.

In the analyzed subgroup, median OS had not been reached at the time of data cutoff and survival analysis (January 2026) (Figure 4), with a total of three deaths recorded.

### 2.7. POEMS

The POEMS syndrome subgroup included 10 patients, with a median age of 52 years (range 32–76 years). Most patients were male (9/10, 90%). All patients were diagnosed according to IMWG criteria [1,11]. The mandatory, major and minor criteria that allowed the diagnosis can be found in Table 3.

At the time of diagnosis, 4/10 patients (40%) had a low performance status, 9/10 patients (90%) had organomegaly, 7/10 (70%) had hepatomegaly, 6/10 (60%) had splenomegaly, and 4/10 (40%) had adenopathy.

Endocrinopathy was detected in 5/10 patients, caused by thyroid dysfunction or hypogonadism. Skin changes were evident in four patients (40%), consisting of hyperpigmentation, skin rigidity, and trophic changes in the nails.

Signs of volume overload were common, occurring in 4/10 patients (40%) and consisting of peripheral edema (4/10 patients), pleural effusion (3/10 patients), and ascites occurring in only one patient (1/10). Other manifestations, such as papilledema, occurred in only one patient (1/10), arterial thrombosis in one patient, and pulmonary hypertension in only one patient.

Significant weight loss was observed in 3/10 patients (30%), and erectile dysfunction in only one patient (10%).

The clinical and electrophysiological evaluation revealed in all patients a predominantly demyelinating sensory–motor polyneuropathy, associated with signs of active denervation on electromyography, suggesting a CIDP-like phenotype. Regarding topographic distribution, 3/10 patients presented simultaneous onset in the upper and lower limbs, while in 7/10 patients the manifestations began exclusively in the lower limbs. Clinically, muscle weakness and paresthesias were the dominant clinical manifestations, both of which were present in all patients.

From a biological point of view, the monoclonal protein isotype in our patient subgroup was: IgG (7/10 patients, 70%), IgA (1/10 patients, 10%), IgM (1/10 patients, 10%), and in one patient, it could not be determined.

The light chain lambda was the most frequently involved (6/10, 60%), with a median value of 35.92 mg/L, and the median value of the monoclonal component was 0.2 g/dL.

The median plasma cell or lymphoplasmacytic infiltrate in MOH was 7%, and FISH testing was performed in 5/10 patients, revealing chromosomal abnormalities such as del17p and ampcr1q in only one patient.

The type of treatment performed in our patient subgroup was quite varied, with most receiving treatment based on proteasome inhibitors, immunomodulators, and cortisone. More details about the treatment are present in Table 2.

IGIV therapy was initiated in seven patients in our subgroup and maintained throughout hematological treatment. In terms of ORR (≥PR), this was recorded in half of our patients (50%). In total, 2/10 patients (20%) recorded a CR response, 1 patient (10%) achieved VGPR, 2 patients achieved PR, and the rest of the patients achieved SD.

Even though only 20% of patients achieved CR, improvement in neurological and systemic symptoms was reported in 60% of patients.

Relapse or disease progression was documented in 2/10 patients (20%), and only one patient died, probably due to cardiovascular complications (pulmonary thromboembolism).

### 2.8. Cryoglobulinemia

The cryoglobulinemia subgroup included five patients, with a median age of 57 years (range 49–68 years) and predominantly female (60%). The median time from symptom onset to diagnosis was 3 months.

Three patients (60%) had IgM MGUS, classified as type II cryoglobulinemia, while two patients (40%) had IgG MGUS, corresponding to type I cryoglobulinemia. The light chain involved was kappa in 4/5 patients (80%), and lambda in only one patient (20%).

The median value of the monoclonal component was 0.3 g/dL, and the median K/L ratio was 3.3 with a median dFLC of 11 mg/L. All five patients were associated with proven HP renal involvement (cryoglobulinemic glomerulonephritis) manifested by nephrotic syndrome. All patients (100%) had skin involvement (cutaneous vasculitis), with one patient (20%) having lower limb amputations. Arthralgia was present in only 2/5 patients (40%), and organomegaly in 1/5 patients (20%).

In the cryoglobulinemia subgroup, electrophysiological investigations could not be performed due to severe skin involvement, including skin ulcerations, or significant edema occurring in the context of nephrotic syndrome related to renal involvement. Therefore, the evaluation of neuropathy in this subgroup was based on clinical examination, with paresthesias being present in all patients (5/5 patients, 100%).

Treatment targeted the hematological clone, consisting of Rituximab-based combinations in the three patients diagnosed with IgM MGUS, while those with IgG MGUS were treated with Daratumumab-based combinations (DRD, D-VCD) (Table 2).

## 3. Discussion

Our study provides a real-world perspective on MGNS, including systemic conditions such as POEMS syndrome and cryoglobulinemia.

The main observation concerns the discrepancy between the small size of the hematologic clone and the severity of organ damage, which supports the MGCS paradigm that therapeutic decisions should be guided by clinical impact [3,4,16].

Data from the literature demonstrate that in MGCS, a small hematologic clone can cause irreversible organ damage [1,2,3,4,16]. Our study also revealed a low clonal burden with a median clonal infiltrate of approximately 8% in MOH and a median component value of approximately 0.3 g/dL.

The prevalence of PN in patients with MGUS is highlighted by numerous studies, ranging from 4% in some studies to 31% in MGUS IgM, compared to 6% in MGUS IgG and 14% in MGUS IgA [8,9,19]. In our cohort, in the entire MGUS group, the prevalence of PN was not rare, being present in 19–20%.

In our cohort, the prevalence of peripheral neuropathy was even higher among patients with IgM MGUS (36.7%) compared to IgG (15.1%), IgA (14.3%), and light chain MGUS (16.7%).

In addition, a relevant statistical correlation was validated between the MGUS subtype and the development of neuropathy (χ^2^(3) = 18.396, *p* < 0.001), with the IgM-MGUS subgroup having a 3.27-fold higher risk compared to the other non-IgM-MGUS subgroups. This finding further supports a potential causal relationship, consistent with the immune-mediated mechanisms described in the neurological literature [8,13].

Also, in our cohort, a greater accidental association between PN and MGUS was more frequent in the IgG subgroup compared to IgM and IgA MGUS where the causal relationship between PN and MGUS appeared to predominate (26.7% vs. 10% in IgM MGUS and 10.7% vs. 3.6% in IgA MGUS). These findings are consistent with the literature, which also suggests that PN is more often an incidental finding in non-IgM MGUS [8,9,19].

Such an association should be considered with greater caution in these subtypes, and in the absence of specific entities such as POEMS syndrome or AL amyloidosis [8].

Taking these data into account, we recommend that electrophoresis and serum protein immunofixation be performed as part of the screening for any peripheral polyneuropathy [7].

Previous population analyses have shown that the presence of peripheral neuropathy in patients with MGUS is associated with an increased risk of mortality, suggesting that neuropathy may be an indicator of frailty or an underlying systemic condition, thus justifying closer monitoring and a multidisciplinary approach to patients with PN and MGUS [9].

In our cohort, the median overall survival was not reached during the follow-up period. The five deaths recorded during the study had heterogeneous causes, predominantly infectious, but also thromboembolic or undetermined. Given the complex clinical profile of the patients, marked by comorbidities, organ involvement, and variable therapeutic exposure, these events should be interpreted with caution. Our data do not allow a direct attribution of an increased risk of mortality to MGNS per se, and the results on overall survival should be analyzed in the context of the small number of patients and the limited follow-up period.

In the non-POEMS MGNS cohort, the heterogeneity of this entity is confirmed [8], although the IgM isotype was predominant, a relevant percentage was represented by IgG or LC.

The distribution of isotypes in our cohort is consistent with the published series, confirming that MGNS is not exclusively an IgM pathology [8].

The literature describes MGNS as a spectrum of neuropathies associated with gammopathies, in which electrophysiological confirmation is essential for phenotype classification [6,7,8]. The significant proportion of CIDP-like phenotypes in our cohort is consistent with the fact that IgM MGUS can mimic CIDP, and differentiation has therapeutic implications, as the response to IVIG or clone-directed therapies may vary depending on the mechanism [4,7].

In our series, anti-MAG neuropathy was identified in a small number of patients, and in the majority of the cases, testing was not performed. The EMG pattern in patients in our cohort with anti-MAG neuropathy was CIDP-like demyelinating PN. In the literature, anti-MAG neuropathy is a subtype of IgM-associated demyelinating neuropathy, in which B-cell-directed therapies are frequently used, particularly rituximab-based therapies, but also BTK inhibitors [21], and the reduction in autoantibodies may require time and/or retreatment, which may explain the delay in clinical and electrophysiological response [8].

Patients with POEMS met the IMWG criteria, and the triad of PN + monoclonal gammopathy + osteosclerotic lesions was consistent. Guidelines emphasize the role of VEGF as a useful diagnostic and monitoring biomarker, but in our cohort, its determination was limited [11]. Our observation that clinical improvement exceeded the hematologic CR rate is consistent with the description of POEMS as a cytokine/VEGF-mediated syndrome, where biological and systemic manifestations may be controlled even without a profound hematologic response [22].

The cryoglobulinemia subgroup presented with universal skin involvement, frequent renal involvement, and nephrotic syndrome with severe complications. The literature shows that type I is more often associated with severe skin manifestations and increased morbidity, which overlaps with the clinical severity observed in our cohort [13,14,15]. Reports and clinical series support the use of proteasome inhibitor-based regimens in refractory or severe monoclonal cryoglobulinemia, especially when the substrate is plasmocytic [13].

Regarding the treatment of patients, according to published data, clinical improvement has frequently exceeded the frequency of profound hematological responses, emphasizing that in MGCS, functional control of organ involvement is a major therapeutic goal, even in the absence of CR [8].

Overall, our research may have practical implications for disease management. This study focuses on the integration of hematological and neurologic approaches in patients with monoclonal gammopathy and peripheral neuropathy, highlighting the importance of distinguishing causal from incidental factors by analyzing clinical and electrophysiological findings, as well as evaluating associated organ involvement, which may help orient both diagnosis and treatment. In this setting, treatment should not be guided solely by hematologic response depth, but also by control of organ dysfunction and neurological improvement, which remain the major therapeutic goals in MGCS.

Our study has limitations due to its retrospective design and small subgroup sizes, which require cautious interpretation of comparative analyses and survival outcomes. However, the long follow-up period and detailed clinical and biological characterization lend robustness to our observations.

## 4. Materials and Methods

This retrospective, single-center study included patients diagnosed with MGNS between January 2015 and December 2025 at the Fundeni Clinical Institute, Bucharest, within the Hematology Department.

During this period, 300 patients with monoclonal gammopathy of undetermined significance (MGUS) were diagnosed, being used as a reference population for the analysis of neuropathy prevalence.

The distribution of MGUS subtypes in the 300 patients was as follows: 199 patients with IgG MGUS, 60 patients with IgM MGUS, 28 patients with IgA, 12 patients with light chain type, and 1 patient with an unknown subtype.

Of these, 35 patients presented neurological involvement, thus meeting the diagnostic criteria for MGNS, Figure 1. These patients constituted the analyzed cohort.

During the same period, 24 patients were identified with monoclonal gammopathy associated with peripheral polyneuropathy, considered incidental, with no evidence to support a causal relationship between monoclonal protein and neurological impairment, being analyzed only in the calculation of the prevalence of MGUS-associated neuropathy.

Also, during the same time interval, 34 patients were diagnosed with histopathologically proven MGRS, with a prevalence of 11.6%. In our cohort, only patients with MGRS who had at least one other organ involvement, either neurological or cutaneous, were included. Patients with AL amyloidosis were excluded.

Several methods were used to diagnose these conditions:

○ Serum protein electrophoresis (sPEP), urinary protein electrophoresis (uPEP), serum immunofixation (sIFE), and urinary immunofixation (uIFE) were performed using agarose gels, followed by immunofixation using Hydragel 4 (Sebia, Évry-Courcouronnes, France).
○ Serum light chain (sFLC) levels were measured using κ and λ reagents on the Optilite turbidimetric system (The Binding Site, Birmingham, UK) and BN ProSpec nephelometer (Siemens Healthineers, Erlangen, Germany).
○ Bone marrow aspiration was performed using K2-EDTA tubes (BD Biosciences, San Jose, CA, USA) to assess the percentage of plasma cells or B lymphocytes on hematoxylin-eosin-stained smears.
○ Bone marrow biopsy sections were stained with hematoxylin-eosin. Immunohistochemical analysis was performed using the Ventana Benchmark Ultra system (Roche Diagnostics GmbH, Mannheim, Germany) with antibodies against CD138 (clone B-A38 RTU, Cell Marque, Rocklin, CA, USA), κ light chain, and λ light chain.
○ The clonality of plasma cells/lymphocytes was assessed by flow cytometry and/or immunohistochemistry on bone marrow biopsy sections [23]. Flow cytometry was performed using the Multiple Myeloma Minimal Residual Disease Kit (MM MRD Kit; Cytognos S.L., Salamanca, Spain; BD Biosciences, San Jose, CA, USA; REF: CYT-MM-MRD8).

Neurological evaluations were performed by neurologists specializing in peripheral neuropathies and included detailed clinical examinations, considering the distribution of motor and sensory symptoms, osteotendinous reflexes, autonomic dysfunction, and the level of functional disability.

Nerve conduction studies (NCSs) and electromyography were available for almost all patients (29/35 patients—82%) suspected of neurological involvement, being performed at diagnosis and subsequently after completion of treatment. The reasons why EMG investigations could not be performed for all patients were either skin lesions of the lower limbs existing at diagnosis in patients with cryoglobulinemia, or significant edema in patients with associated MGRSs and consecutive nephrotic syndromes.

Depending on the mechanism of neuropathy, it was classified as axonal, demyelinating, or mixed, in accordance with electrophysiological standards [7]. Depending on the type of fiber predominantly affected, it was classified as sensory, motor, or sensory–motor [7].

Serum anti-MAG IgM antibody testing was performed in 10/35 patients (28.5%) using indirect immunofluorescence techniques, with results reported qualitatively (positive/negative) according to laboratory criteria.

The diagnosis of MGNS was made based on the following criteria [1,7]:

○ The presence of monoclonal gammopathy.
○ Peripheral neuropathy confirmed by clinical and/or electrophysiological methods.
○ The exclusion of other common causes of neuropathy (such as diabetes mellitus, vitamin B12 deficiency, chronic alcoholism, autoimmune disorders, disc herniation, etc.).
○ Indicators suggesting an autoimmune or paraneoplastic mechanism (anti-MAG antibodies, cryoglobulins present, response to clone-targeted treatments).
○ A peripheral nerve biopsy was performed in only one patient in our group, in the context of persistent clinical suspicion and as necessary to determine the MGUS-PN causal relationship.
○ A whole-body low dose CT was performed in all patients with IgA/IgG MGUS and LC to exclude osteolytic lesions that could indicate a diagnosis of multiple myeloma (MM) and to ensure appropriate imaging staging.
○ A thoracic–abdomen–pelvic CT with contrast substance was performed for all patients with IgM MGUS, POEMS syndrome, and cryoglobulinemia in order to identify any specific lymphadenopathy and organomegaly.
○ PET-CT was performed, when possible, in selected cases of POEMS syndrome; particularly to detect active metabolic osteosclerotic lesions, assess disease extent, and guide therapeutic approaches.

The criteria for POEMS diagnosis used were those according to the IMWG [11].

The neurological response to treatment was assessed clinically and electrophysiologically when available, being classified as present if there was clinical improvement or improvement evident on ENMG, stable if there was no neurological progression, and absent if there were no signs of improvement or stabilization of the neurological disease.

The hematological response was determined based on IMWG criteria [20].

The diagnosis of MGRS for patients included in our cohort was established based on currently accepted criteria involving the existence of renal damage confirmed histopathologically by renal biopsy and the demonstration of monoclonal nature by immunohistochemistry or immunofluorescence, as well as identification of the responsible hematological clone [16].

Statistical analyses were performed using IBM SPSS Statistics, version 27.0 (IBM Corp, Armonk, NY, USA) and Microsoft Excel, version 16.78.3 (Microsoft Corp., Redmond, WA, USA). Overall survival (OS) was defined as the time interval from diagnosis to death from any cause or last evaluation for living patients. OS was evaluated through the Kaplan–Meier technique, and subgroup comparisons were performed with the log-rank test. The median duration of follow-up was determined by using the reverse Kaplan–Meier approach. For prevalence studies, categorical variables were presented as frequencies and percentages, and were compared between categories using the χ^2^ (Pearson) test. In situations where the conditions required for the use of the χ^2^ test were not fully met (e.g., expected frequencies <5), the Monte Carlo test was performed. For 2 × 2 comparisons, the Fisher test was used.

Quantification of the association of peripheral polyneuropathy according to MGUS subtype (IgM vs. non-IgM) was calculated using odds ratio (OR) with a 95% confidence interval (CI).

Due to the limited sample size, the subgroup analyses were considered exploratory.

## 5. Conclusions

This single-center retrospective study provides a detailed real-world analysis of the clinical, biological, and evolutionary aspects of monoclonal gammopathies affecting the main nervous systems and skin. The results obtained demonstrate that monoclonal gammopathies are active clinical entities, characterized by a significant separation between the sizes of the hematological clones and the severity of organ involvement.

The conclusions of this study demonstrate the need for awareness among neurologists and hematologists, as another strategy is needed in the management of MGNS, focusing on the impact of the monoclonal protein on the clinical condition of patients and not just on traditional hematological markers. To create uniform guidelines for diagnosis, follow-up, and treatment, as well as to identify predictive characteristics that may support personalized therapies in these rare but clinically important cases, further research and multicenter databases are required.

## Figures and Tables

**Table 1:** General characteristics of included patients.

Characteristic	Value	Percent
Number of patients, *n*	35	
Age, median (range) years	59 (32–86)	
Male sex, *n* (%)	21	60%
Follow-up duration, median (range), months	28.3	
Deaths during follow-up, *n* (%)	5	14.2%
Median time to diagnosis (range) months	11 (0–271)	
Study period (interval)	2015–2025	
MGCS subtype, *n* (%)		
- MGNS (including POEMS syndrome) - Cryoglobulinemia	30 5	85% 15%
Monoclonal protein isotype, n (%)		
- IgM - IgG - IgA - LC - unknown	16 13 3 2 1	45.7% 37.1% 8.5% 5.71% 2.85%
Light chain type, *n* (%)		
- Kappa - Lambda - unknown	20 14 1	57% 40% 2.85%
Associated MGRS, *n* (%) - glomerulonephritis with monoclonal immunoglobulin deposits (PGNMID) - immunotactoid glomerulonephritis - light chain proximal tubulopathy - cryoglobulinemic glomerulonephritis	11 3 1 2 5	31.42% 8.5% 2.85% 5.71% 15%
Type of clone - Plasma cells - Lymphoplasmacytic - Lymphocytes - unknown	21 8 5 1	60% 22.8% 14.2% 2.85%
Median bone marrow clonal infiltration (%)		8%
Median M-protein value, g/dL	0.3	
Median LC involved, mg/L	31.85	
Median dFLC, mg/L	11	
Median LDH (U/L)	250	

**Table 2:** Hematological treatment in the entire cohort. ASCT, autologous stem cell transplantation; BR, bendamustine–rituximab; DRC, dexamethasone–rituximab–cyclophosphamide; DRD, daratumumab–lenalidomide–dexamethasone; D-VCD, daratumumab–bortezomib–cyclophosphamide–dexamethasone; PI, proteasome inhibitor; IMiDs, immunomodulatory drugs; KRD, carfilzomib–lenalidomide–dexamethasone; POEMS, polyneuropathy, organomegaly, endocrinopathy, monoclonal protein, and skin changes; RAD, rituximab–adriamycin–dexamethasone; RD, lenalidomide–dexamethasone; R-CHOP, rituximab–cyclophosphamide–doxorubicin–vincristine–prednisone; R-CVP, rituximab–cyclophosphamide–vincristine–prednisone; RT, radiotherapy; VCD, bortezomib–cyclophosphamide–dexamethasone; VRD-lite, bortezomib–lenalidomide–dexamethasone lite.

Hematological Treatment	*n* (%)
MGNS (excluding cryoglobulinemia and POEMS syndrome)	
Based on anti-CD38 antibody	3 (17.6%)
. DRD	2 (11.7%)
. D-VCD	1 (5.8%)
Based on anti-CD20 antibody	8 (47.05%)
. BR	1 (5.8%)
. DRC	3 (17.6%)
. R-Ibrutinib	1 (5.8%)
. R-Cyclophosphamide	2 (11.7%)
. R-CVP	1 (5.8%)
Based on PI therapy and/or IMIDs	4 (23.5%)
. KRD	1 (5.8%)
. VCD	1 (5.8%)
. RAD	1 (5.8%)
. RD	1 (5.8%)
Methylprednisolone pulse	2 (11.7%)
ASCT	2 (11.7%)
POEMS syndrome subgroup	
Based on IP treatment	6 (60%)
. KRD	5 (50%)
. VRD-lite	1 (10%)
Based on anti-CD20 antibody	2 (20%)
. R-Bendamustine	1 (10%)
. R-CHOP	1 (10%)
Cyclophosphamide-Dexamethasone	1 (10%)
ASCT	4 (40%)
RT	4 (40%)
Cryoglobulinemia	
Based on anti-CD20 antibody	3 (60%)
. BR	1 (20%)
. R-CVP	1 (20%)
. R-Cyclophosphamide-Dexamethasone	1 (20%)
Based on anti-CD38 antibody	2 (40%)
. DRD	1 (20%)
. D-VCD	1 (20%)

**Table 3:** POEMS diagnosis criteria in our group.

Criteria	*n* (%)
Mandatory criteria	
(1) Demyelinating polyneuropathy (2) Monoclonal plasma-cell-proliferative disorder	10 (100%) 10 (100%)
- Kappa - Lambda - Unknown	3 (30%) 6 (60%) 1 (10%)
Major criteria	
(1) Sclerotic bone lesions	10 (100%)
- >2 site	7 (70%)
(2) VEGF elevation (3) Castleman disease	4 (40%) 1 (10%)
Minor criteria (1) Organomegaly (2) Extravascular volume overload (3) Endocrinopathy (4) Skin changes (5) Papilledema (6) Thrombocytosis/polycythemia	9 (90%) 4 (40%) 5 (50%) 4 (40%) 1 (10%) 4 (40%)

## Data Availability

The original contributions presented in this study are included in the article. Further inquiries can be directed to the corresponding author.

## Abbreviations

The following abbreviations are used in this manuscript:

MGCS	Monoclonal Gammopathy of Clinical Significance
MGUS	Monoclonal Gammopathy of Undetermined Significance
Mo Ig	Monoclonal Immunoglobulin
MM	Multiple Myeloma
MGRS	Monoclonal Gammopathy of Renal Significance
MGNS	Monoclonal Gammopathy of Neurological Significance
MGOS	Monoclonal Gammopathy of Ocular Significance
MGSS	Monoclonal Gammopathy of Cutaneous Significance
MGTS	Monoclonal Gammopathy of Thrombotic Significance
PN	Peripheral Neuropathy
AL-Amyloidosis	Light-Chain Amyloidosis
POEMS	*Polyneuropathy*, *organomegaly*, *endocrinopathy*, *M-protein*, *skin changes*
DADS-M	Distal Acquired Demyelinating Symmetric
CANOMAD	chronic ataxic neuropathy, ophthalmoplegia, immunoglobulin M [IgM] paraprotein, cold agglutinins, and disialosyl antibodies
SLONM	Sporadic late onset nemaline myopathy
VEGF	Vascular Endothelial Growth Factor
CIDP	Chronic inflammatory demyelinating polyneuropathy
PGNMID	Proliferative glomerulonephritis with monoclonal immunoglobulin deposit
LCPT	Light chain proximal tubulopathy
OS	Overall Survival
ORR	Overall Response Rate
CR	Complete Response
VGPR	Very Good Partial Response
PR	Partial Response
SD	Stable Disease
dFLC	difference between involved and uninvolved serum free light chains
LDH	Lactate dehydrogenase
LC	Light Chain
EMG	Electromyography
MAG	Myelin-associated glycoprotein
BR	Bendamustine-Rituximab
DRC	Dexamethasone-Rituximab-Cyclophosphamide
R-IBRUTINIB	Rituximab-Ibrutinib
R-CYCLOPHOSPHAMIDE	Rituximab-Cyclophosphamide
DRD	Daratumumab-Lenalidomide-Dexamethasone
D-VCD	Daratumumab-Bortezomib-Cyclophosphamide-Dexamethasone
KRD	Carfilzomib-Lenalidomide-Dexamethasone
VCD	Bortezomib-Cyclophosphamide-Dexamethasone
RAD	Lenalidomide-Adriamycin-Dexamethasone
RD	Lenalidomide-Dexamethasone
ASCT	Autologous Stem Cell Transplant
IMIDS	Immunomodulatory Drugs
PI	Proteasome Inhibitors
IMWG	International Myeloma Working Group
MOH	Bone Marrow
VRD-LITE	Bortezomib-Lenalidomide-Dexamethasone reduced intensity protocol
R-CHOP	Rituximab-Cyclophosphamide-Adriamycin-Vincristine-Dexamethasone
IGIV	Immunoglobulin Intravenous
BTK	Bruton Tyrosine Kinase
uPEP	urinary protein electrophoresis
uIFE	urinary protein electrophoresis
sIFE	serum immunofixation
sFLC	Serum light chain
sPEP	Serum protein electrophoresis
NCS	Nerve Conduction Studies
PET-CT	Positron Emission Tomography—Computed Tomography
CT	Computed Tomography
